# Tag-based next generation sequencing: a feasible and reliable assay for *EGFR* T790M mutation detection in circulating tumor DNA of non small cell lung cancer patients

**DOI:** 10.1186/s10020-019-0082-5

**Published:** 2019-04-27

**Authors:** Mariella Dono, Giuseppa De Luca, Sonia Lastraioli, Giorgia Anselmi, Maria Giovanna Dal Bello, Simona Coco, Irene Vanni, Francesco Grossi, Antonella Vigani, Carlo Genova, Manlio Ferrarini, Jean Louis Ravetti, Simona Zupo

**Affiliations:** 1 Molecular Diagnostic Unit, IRCCS Ospedale Policlinico San Martino, L.go R. Benzi 10, 16132 Genova, Italy; 2 Pathology Department IRCCS Ospedale Policlinico San Martino, Genova, Italy; 3 Lung Cancer Unit, IRCCS Ospedale Policlinico San Martino, Genova, Italy; 4 UOC Oncologia Medica, Fondazione IRCCS Ca’ Granda, Ospedale Maggiore Policlinico, Milan, Italy; 5 Oncology Unit, Ospedale S. Andrea, La Spezia, Italy; 6 UOC Oncologia Medica, IRCCS Ospedale Policlinico San Martino, Genova, Italy; 7 DIMES, Anatomy Section, University of Genova, Medical School, Genova, Italy

**Keywords:** Circulating tumor DNA, Liquid biopsy, NSCLC, EGFR TKIs, T790M resistance mutation, Molecular tag, Next generation sequencing, C797S

## Abstract

**Background:**

The demonstration of *EGFR* T790M gene mutation in plasma is crucial to assess the eligibility of Non Small Cell Lung Cancer (NSCLC) patients, who have acquired resistance to first or second generation Tyrosine Kinase Inhibitors (TKIs), to receive a subsequent treatment with osimertinib. Since circulating tumor DNA (ctDNA) is present in very low amounts in plasma, high sensitive and specific methods are required for molecular analysis.

Improving sensitivity of T790M mutation detection in plasma ctDNA enables a larger number of NSCLC patients to receive the appropriate therapy without any further invasive procedure.

**Methods:**

A tag-based next generation sequencing (NGS) platform capable of tagging rare circulating tumor DNA alleles was employed in this study for the identification of T790M mutation in 42 post-TKI NSCLC patients.

**Results:**

Compared to Real Time PCR, tag-based NGS improved the T790M detection rate (42.85% versus 21.4%, respectively), especially in those cases with a low median mutation abundance (i.e. 0.24, range 0.07–0.78). Moreover, the tag-based NGS identified *EGFR* activating mutations more efficiently than Real Time PCR (85.7% versus 61.9% detection rate, respectively), particularly of the L858R variant type (0.06–0.75 mutation abundance range). Patients in whom the T790M mutation was detected in plasma, achieved an objective response to osimertinib (9/14, 64.28%).

**Conclusions:**

Tag-based NGS represents an accurate and sensitive tool in a clinical setting for non-invasive assessment and monitoring of T790M variant in NSCLC patients.

**Electronic supplementary material:**

The online version of this article (10.1186/s10020-019-0082-5) contains supplementary material, which is available to authorized users.

## Background

The current standard work-up of Non Small Cell Lung Cancer (NSCLC) patients includes the search for sensitizing mutations of *EGFR* gene (Sharma et al. 2007; Riely et al. 2006; Rosell et al. 2010; Mok et al. 2009) that allowed identification of patients eligible for treatment with an EGFR tyrosine kinase inhibitor (TKI) (Singh & Jadhav 2018). Most patients respond to first and second-generation EGFR TKIs, such as gefitinib, erlotinib and afatinib, but acquired resistance is likely to occur, leading to disease progression. *EGFR* T790M substitution has been indicated as the prevalent molecular event involved and occurs in approximately 50–60% of the cases developing TKI resistance (Yu et al. 2013; Hata et al. 2013; Sequist et al. 2011; Oxnard et al. 2011; Cross et al. 2014). Osimertinib is a third-generation EGFR TKI, designed to overcome resistance due to T790M and representing the current standard treatment for advanced, T790M-positive NSCLC patients progressing after first or second- generation EGFR TKI (Cross et al. 2014; Ramalingam et al. 2018). However, more recently the U.S. Food and Drug Administration (FDA) has approved the use of osimertinib also in first line for advanced NSCLC harboring common *EGFR* mutations (Mok et al. 2017).

Although T790M can be identified through a new biopsy of the progressing neoplasm, this procedure may be challenging as well as stressful for the patient, and could potentially lead to complications. Several studies have demonstrated the feasibility of assessing *EGFR* mutational status on circulating cell-free DNA (cfDNA) from plasma (Douillard et al. 2014; Sorensen et al. 2014; Sundaresan et al. 2016; Vanni et al. 2015). The cfDNA is becoming a reliable alternative source to tumor DNA, although the sensitivity of methods using cfDNA is generally lower (Ramalingam et al. 2018; Vanni et al. 2015; Luo et al. 2014; Oxnard et al. 2016). This approach is non-invasive, does not pose limitations to repeated sampling, and provides a sufficiently accurate assessment of intra and inter-tumor heterogeneity (Sundaresan et al. 2016; Murtaza et al. 2013; Diaz and Bardelli, 2014). Because circulating cell-free tumor-derived DNA (ctDNA) is diluted out with normal DNA, ctDNA analysis is technically challenging requiring both sensitivity and accuracy (Murtaza et al. 2013). The current methods for the detection of plasma T790M in clinical practice include digital PCR (dPCR) techniques, Real Time PCR assays and Next Generation Sequencing (NGS) (Thress et al., 2015a; Bartels et al. 2017; Kim et al. 2013; Mayo-de-las-Casas et al. 2017). Variable T790M detection rates have been reported ranging between 31 and 66% for BEAMing (beads, emulsion, amplification and magnetics) digital PCR; 18–52% for droplet digital PCR (ddPCR) and 22–30% for common Real Time PCR assays (Luo et al. 2014; Mayo-de-las-Casas et al. 2017). Improving the reliability of T790M detection in cfDNA would represent a significant achievement, as it would permit the access to an effective therapeutic agent to a larger number of patients in the absence of repeated tissue biopsies. Here, we have studied a commercial NGS panel using molecular tagging of the DNA alleles present in the plasma, and compared the results obtained with those by Real Time PCR.

## Material and methods

### Patients

Plasma samples of 42 patients with NSCLC were collected between 2016 and 2018. This cohort included patients with a histologic diagnosis of advanced NSCLC harboring sensitizing *EGFR* mutations, who experienced disease progression while on treatment with a first or second-generation EGFR TKI. This study was approved by the Ethics Committee of Liguria Region (Italy) (P.R.273REG2016) and conducted in compliance with the principle of the Declaration of Helsinky; a written informed consent was acquired from all patients. The relevant clinical characteristics of the patients are summarized in (Additional file 1: Table S1).

### Samples collection

Peripheral blood (12–18 mL) was collected into EDTA-containing tubes. Plasma was obtained by two centrifugation rounds at 1600 x g and 3000 x g, both for 10 min at 4 °C within 2 h from collection.

Post-TKI tumor tissue was obtained from 15/42 patients undergoing biopsy for T790M analysis. The Multiplex I cfDNA Reference Standards at 5, 1, 0.1 and 0% allelic frequencies (HD780, Horizon Diagnostics) mimicking human fragmented cfDNA (average 160 bp), were used to evaluate the performance of Oncomine™ Lung cfDNA Assay for sensitivity and specificity.

### DNA extraction and *EGFR* mutations detection by Real Time PCR

Plasma cfDNA was extracted from 3 mL of plasma using the QIAamp Circulating Nucleic Acid kit (Qiagen, Hilden, Germany) according to the manufacturer’s instructions. Formalin-fixed paraffin-embedded (FFPE) tumor tissue sections (5 μm thickness) were used for genomic DNA extraction with QIAamp FFPE tissue kit (Qiagen). A dedicated kit was used for the extraction of DNA from cytological slides according to the manufacturer’s instruction (PinPoint Slide DNA Isolation System, Zymo Research, Euroclone, Milano, Italy). In both tissues and cytological samples, tumor enrichment was performed by macrodissection of areas containing at least 50% of neoplastic cells.

*EGFR* mutations in cfDNA and tissue/cytological samples were detected with Easy EGFR Real Time PCR (Diatech Pharmacogenetics, Jesi, Italy). For a limited number of plasma samples (10/42) a Real Time PCR with a different chemistry was employed, PANAMutyper R EGFR (Panagene, Bioclarma, Torino, Italy). Both the assays were developed to achieve selective amplification of mutated allele and suppression of wild type DNA. Internal Controls (IC) were present within the PCR reactions and amplified together with the sample to check purity and concentration of the DNA. Then, manufacturer’s instructions were followed to validate each Real Time test by checking the parameters indicated (Cycle threshold (Ct) values of control gene amplification of DNA samples and Ct values of IC amplification).

### Procedures for tag-based NGS testing

#### cfDNA extraction

cfDNA was isolated from 1.4–3 mL of plasma using MagMAX™ Cell-Free DNA Isolation Kit according to manufacturer’s instructions (ThermoFisher Scientific, Carlsbad, CA) and quantified using the Qubit® dsDNA HS Assay Kit on the Qubit 3.0 fluorometer (ThermoFisher). The purity and quantity of DNA size fragments was analyzed by the Agilent High Sensitivity DNA Analysis Kit (Agilent Technologies, Santa Clara, CA, USA) using Bioanalyzer 2100 instrument (Agilent Technologies).

#### Library preparation and quantification

Targeted libraries were amplified using Oncomine™ Lung cfDNA Assay (ThermoFisher). The assay includes 35 amplicons covering 169 key hotspot mutations in 11 genes (*ALK*, *BRAF*, *EGFR*, *ERBB2*, *KRAS*, *MAP2K1*, *MET*, *NRAS*, *PIK3CA*, *ROS1*, and *TP53*) (Additional file 2: Table S2). Patients cfDNAs (range 6–52 ng per reaction) were employed to prepare manually targeted libraries following manufacturer’s instructions.

Briefly, each cfDNA molecule was assigned with unique molecular tag through a first PCR reaction in a Veriti thermal cycler (Applied Biosystems™, Foster City, CA) and subsequently, tagged library fragments were amplified in a second round of PCR to produce independent barcoded libraries. Libraries were purified using Agencourt™ AMPure™ XP beads (Beckman Coulter, Milano, Italy).

For library quantification, a qPCR (with Ion Library TaqMan Quantitation Kit, ThermoFisher) was performed and run on StepOne instrument (Applied Biosystems™).

#### Template preparation, sequencing and data analysis

For the template preparation, 4 libraries (diluted to 50 pM) were multiplexed, and sequencing was subsequently performed using Ion 520™ chip (5 × 10^6^ of reads capability), loaded on Ion Chef™ Instrument and run on Ion S5 instrument (ThermoFisher). The sequencing reads were aligned and mapped to the reference human genome sequence (hg19) using the Torrent Mapping Alignment Program (TMAP). The plugin Torrent Variant Caller (TVC, version 5.8, ThermoFisher) with specific parameters for liquid biopsy inside the JSON (JavaScript Object Notation) file was run in order to detect and report only the variants hotspot alleles that meet criteria for calling*,* i.e. a call was made when at least two family tags shared an identical mutation (corresponding to two independent single mutated DNA alleles) and each family tag displayed at least 3 reads. Optimal results were obtained with Median Read Coverage > 25,000 and Median Molecular Coverage > 2,500 (Oncomine™ cfDNA Assays Part III: Variant Analysis User guide).

Review of all the hotspots calls was performed by uploading each Variant Call Format (VCF) file on IGV (Integrative Genomics Viewer, Cambridge MA, https://software.broadinstitute.org/software/igv/home).

## Droplet digital PCR (ddPCR)

Validation of *EGFR* T790M mutations was performed by QX200 ddPCR™ System (Bio-Rad Laboratories, Inc., Hercules, CA, USA) using ddPCR Mutation Detection Assays (FAM-Mutation assay: dHsaCP2000019 and HEX-wild type assay: dHsaCP2000020). For each PCR reaction 10 ng of FFPE DNA or 5–10 μl of cfDNA obtained by QIAamp (Qiagen) was amplified according to the ddPCR mutation protocol (Oxnard et al. 2014). Each PCR run, including samples (FFPE DNA and/or cfDNA in 2–4 replicates to screen at least 3.000 genomes per case) and controls (wild type, T790M-positive and no template controls) was analyzed using QuantaSoft analytical software package (Bio-Rad Laboratories). Threshold was determined according to the signals of no template, wild-type DNA and T790M-positive control. The allele fraction for each sample was calculated as merged of replicates, and the positive sample was called when at least 3 FAM-positive droplets were detected. The limit of detection (LOD) of the ddPCR T790M assay was initially determined and achieved values of 0.1% when at least 10 ng of DNA were analyzed. Since a number of studies described false-positive *EGFR*T790M mutation in FFPE NSCLC tumors (Ye et al., 2013; Do et al., 2017), we set a cut-off > 0.5% by “in house” experiment using 22 FFPE normal tissue samples (Additional file 3: Table S3).

## Statistical analysis

Statistical evaluation of the data in this study was performed using GraphPad Prism version 6 software and XLSTAT (v.19.03.44845). Threshold for statistical significance was considered to be *P* < 0.05.

## Results

### Analytical validation of Oncomine™ Lung cfDNA assay

Sensitivity testing was initially performed starting from 30 ng cfDNA of each Multiplex I cfDNA Reference Standard at 5, 1, 0.1 and 0% mutation frequencies and analyzing specifically 4 different *EGFR* hotspots, that is E746_A750del, L858R, T790M and V769_D770ins (Table 1). The tag-based NGS detected all *EGFR* mutations down to the 0.1% allele frequency with high concordance between the measured allele frequencies with those expected for each reference cfDNAs (Table 1). Since the mutant DNA copies for L858R resulted underestimated compared to mutant copies found for the other three variants (Table 1), we checked reproducibility of L858R variant call in critical samples (i.e. those with input cfDNA < 30 ng) and tested the assay using 20 ng of the reference standard at 0.1% mutated allele frequency. A positive call for the L858R mutation was achieved with the minimum allele molecular coverage, i.e. two molecular tags. In contrast, E746_A750del call was missed, although it can still be visualized on IGV and identified by a single molecular tag. No false positive call were observed with the test cfDNA containing wild type *EGFR* gene (0% mutated allele frequency) only, even when the VCF was visualized on IGV software.

**Table 1:** Analytical testing of tag-based NGS

RS	cfDNA input (ng)	Library (pM)	Median Read Cov	Median Mol Cov	*EGFR* gene variants	VAF (%)	LOD (%)	Allele Mol Cov	Tot Read Cov
HD780 (5%)	30	430	53,562	4365	E746_A750delELREA	5.81	0.05	265	62,561
					V769_D770insASV	4.17	0.05	220	47,935
					T790M	5.46	0.05	272	73,309
					L858R	5.02	0.1	119	56,740
HD780 (1%)	30	408	27,829	3614	E746_A750delELREA	0.91	0.05	37	34,718
					V769_D770insASV	0.85	0.05	34	23,487
					T790M	0.91	0.05	40	37,057
					L858R	1.15	0.1	25	29,792
HD780 (0.1%)	30	450	28,269	3778	E746_A750delELREA	0.14	0.05	6	35,994
					V769_D770insASV	0.1	0.05	4	23,378
					T790M	0.11	0.05	5	33,633
					L858R	0.09	0.1	2	27,703
HD780 (0%)	30	570	32,314	2353	E746_A750delELREA	0	nd	0	
					V769_D770insASV	0	nd	0	
					T790M	0	nd	0	
					L858R	0	nd	0	
HD780 (0.1%)	20	705	22,117	2734	E746_A750delELREA	0.03	0.05	1	29,492
					V769_D770insASV	0.1	0.05	3	20,616
					T790M	0.19	0.05	6	28,438
					L858R	0.13	0.1	2	19,308

*RS* Reference Standard, *Median Read Cov* Median Read Coverage, *Median Mol Cov* Median Molecular Coverage, *VAF* Variant Allele Frequency, *LOD* Limit of Detection, *Allele Mol Cov* Allele Molecular Coverage, *Tot Read Cov* Total Read Coverage, *nd* not detected, *HD* Horizon Discovery

### Comparison of sensitizing and T790M *EGFR* mutations detected by Real Time PCR and tag-based NGS

The plasma cfDNA from a cohort of 42 patients progressing while under TKI was tested by Real Time PCR for *EGFR* mutations. The same samples were subsequently re-tested using Oncomine™ Lung cfDNA Assay and the results of the two technologies compared.

**Fig. 1:**
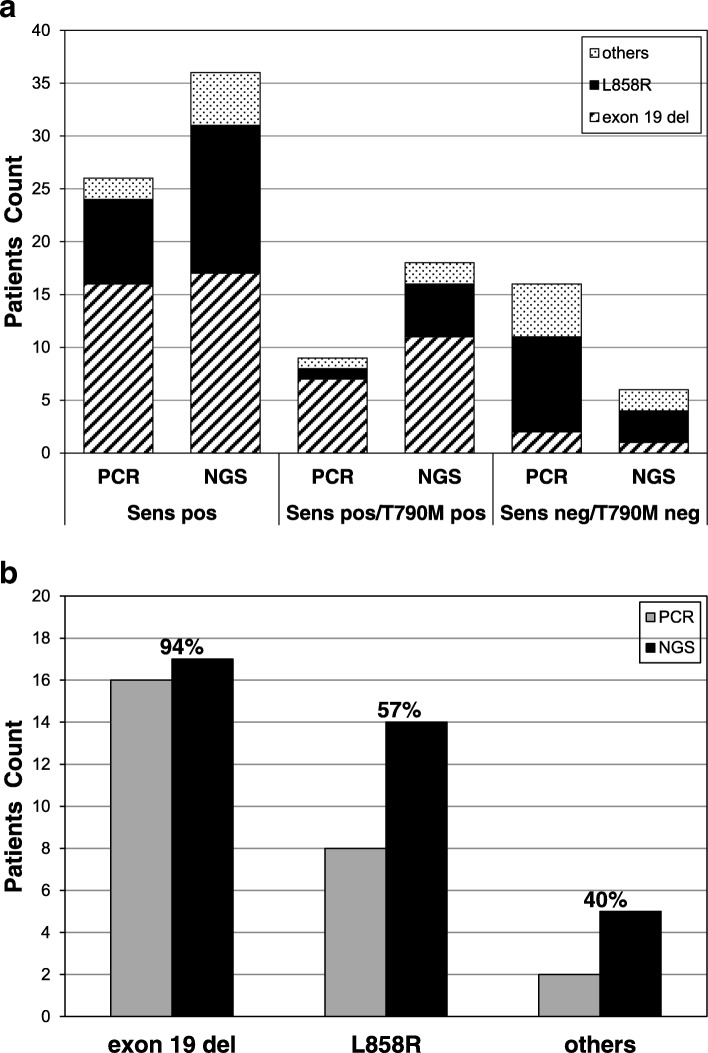
*EGFR* mutations in 42 post-TKI NSCLC patients. (**a**) Distribution of the various *EGFR* mutations types in the 42 patients according to Real Time PCR (PCR) and tag-based NGS (NGS). The y-axis shows patients count according to different mutation patterns detected by the two platforms. (**b**) Coincident Rate between Real Time PCR (grey bars) and tag-based NGS (black bars) according to the different *EGFR* mutations types found. The y-axis indicated the number of cases concordant for the specific variation with both Real Time PCR and tag-based NGS and corresponding percentages are indicated within the histogram. PCR, Real Time PCR; NGS, tag-based NGS; del, deletions; Sens, sensitizing; pos, positive; neg, negative

Using Real Time PCR, 26/42 (61.9%) cfDNA samples displayed the initial sensitizing *EGFR* mutation seen at the diagnosis in the primary tumor and 9/42 (21.4%) also showed a concurrent T790M mutation, whereas the remaining cases (16/42, 38.1%) resulted negative for both mutations (Fig. 1a, Additional file 4: Table S4). Eight out of nine T790M-positive plasma samples by Real Time PCR were also exon 19 deletion positive, while only one case had a L858R co-occurring sensitizing mutation.

According to the above data, the samples with concurrent sensitizing *EGFR* mutations and the T790M substitution were 9/26 (34.6%), a finding consistent with the current reports for Real Time PCR assays (Thress et al., 2015a; Mayo-de-las-Casas et al. 2017).

When tag-based NGS was employed on the same cfDNA samples, the cases positive for the original sensitizing *EGFR* mutation were 36/42 (85.7%), and those harboring the T790M resistance mutation were 18/42 (42.85%) (Fig. 1a, Additional file 4: Table S4). All of the latter cases also had the original sensitizing mutation; therefore, of the 36 cases with a sensitizing mutation, 50% (18/36) also had the T790M mutation, in line with data reported for the detection of resistance mutation in post-TKI tissues (Yu et al. 2013; Hata et al. 2013; Sequist et al. 2011; Oxnard et al. 2011). Among the 18 T790M-positive cases, 11 cases harbored the exon 19 deletion, 5 cases had the L858R mutation and two an unusual *EGFR* mutations, i.e. one had the rare A763_Y764insFQEA exon 20 insertion and one the G719C exon 18 substitution (Additional file 4: Table S4).

Most of tag-based NGS-positive and Real Time PCR-negative cases for sensitizing *EGFR* mutations (6/10 cases) had the L858R mutation, leading to the conclusion that the coincidence rate between the two methods was of 94% for exon 19 deletions and 57 and 40% for the L858R and uncommon *EGFR* mutations, respectively (Fig. 1b).

No difference was observed in each patient regarding the original *EGFR* sensitizing mutation between the primary tumor tissue and the plasma samples at progression with either the Real Time PCR or the tag-based NGS test, indicating that the specificity of both methodologies was 100% (Additional file 4: Table S4).

### Characterization of *EGFR* allelic fraction detected by tag-based NGS

**Fig. 2:**
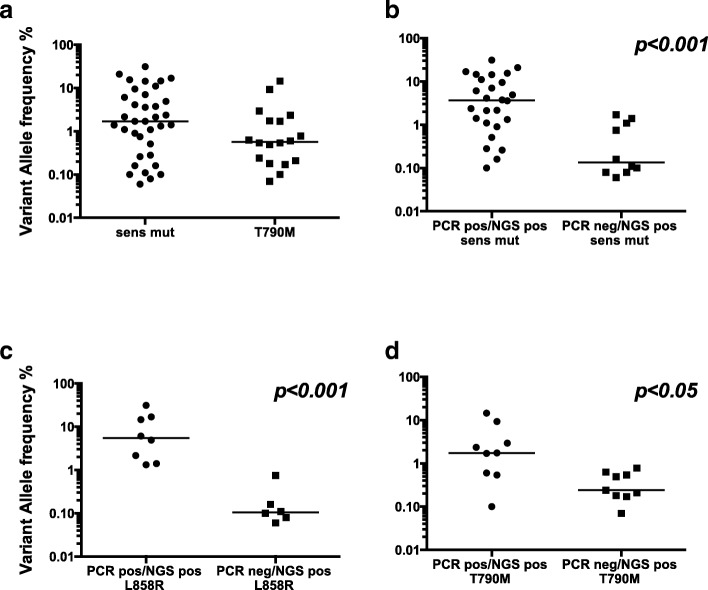
Variant allele frequency (%) in plasma. Sensitizing *EGFR* mutations (*n* = 36 cases) and T790M mutations (*n* = 18 cases) were determined in plasma by tag-based NGS and are reported as variant allele frequency percentage (%) (**a**) Variant allele frequency (%) for *EGFR* mutations determined by tag-based NGS in two patients groups classified as positive or negative for the sensitizing mutation of *EGFR* based on both (black circles) or one (black squares) the methods employed in the study (**b**) Results of tests similar to those in b except that the L858R and T790M mutations were measured in (**c**) and (**d**), respectively. Each dot represents one patient. Solid lines represent median values. Statistical *P* values were derived from a Mann-Whitney test. PCR, Real Time PCR; NGS, tag-based NGS; sens mut, sensitizing mutations; pos, positive; neg, negative

Subsequently, we investigated the proportion of mutant *EGFR* alleles (expressed as variant allele frequency, VAF) present in each patient. First of all, no relationship was found between the whole cfDNA (range 2.8–277 pg/μl of plasma) and the mutational load assessed on *EGFR* gene by tag-based NGS (Additional file 5: Figure S1). Second, when sensitizing *EGFR* mutations were considered (36 cases), the VAF median percentage was 1.705, with a 0.06–31.3 range (Fig. 2a). In detail, the VAF median value of the 10 cases that were Real Time PCR-negative/tag-based NGS-positive for sensitizing *EGFR* mutations, was significantly lower compared to that of the 26 cases that were *EGFR* positive with both methods (0.135 vs 3.68, respectively; *p* = 0.0002 Mann-Whitney test, Fig. 2b). These data indicate that tag-based NGS detects *EGFR* mutations present at low-frequency in cfDNA. Third, the majority of these low frequency *EGFR* mutations (6/10, 60%), were observed among cases harboring the L858R-type mutation (median VAF 0.105, range: 0.06–0.75). Conversely, the median VAF of cases found L858R positive with both Real Time PCR and tag-based NGS was definitely higher, i.e. 5.49 (Fig. 2c, p = 0.0002, Mann-Whitney test).

Fourth, the T790M allele frequencies of the 18 positive cases were shifted towards lower values (median VAF 0.57, range 0.07–14.47) compared to those of *EGFR* mutations (Fig. 2a and d, Del Re et al. 2017). Again, there was a statistically significant difference between the median VAF value of the 9 cases found T790M-positive by tag-based NGS only and that of the 9 cases that resulted positive by both technologies (0.24 and 1.74 respectively).

### Orthogonal validation of T790M by ddPCR

**Fig. 3:**
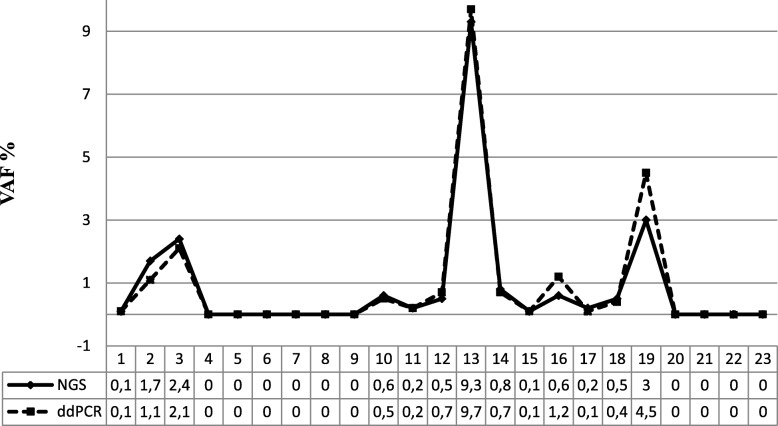
T790M detection comparison between tag-based NGS and ddPCR. Variant Allele Frequencies detected by tag-based NGS (black line) and ddPCR (dashed line) for 23 plasma samples are shown. NGS, tag-based NGS; ddPCR, droplet digital polymerase chain reaction; VAF, Variant Allele Frequency

26/42 patients (10 T790M-negative and 16 T790M-positive cases by tag-based sequencing) also were tested for the T790M mutation by the ddPCR assay. In three cases, classified as T790M-positive by tag-based NGS, the ddPCR test was unsuccessful due to low cfDNA quantity available. These were excluded from the comparative analyses. Thirteen of the remaining 23 cases, that were classified as T790M-positive by the tag-based NGS, were confirmed to be positive by ddPCR with a very similar VAF, likewise the 10 cases classified as negative by the tag-based NGS also were confirmed to be negative by ddPCR (Fig. 3).

### Comparative analyses of *EGFR* mutations in plasma and post-TKI tissues

Post-TKI specimens from 15 patients were tested for *EGFR* mutations by Real Time PCR and results compared with those of cfDNA testing by tag-based NGS (Table 2).

**Table 2:** Comparison of *EGFR* mutational status between plasma and post-TKI tissue samples

*Patient N.*	*EGFR* mutation in post-TKI Plasma		*EGFR* mutation in post-TKI Tissues		Tissue obtained by	Tumor source
	Sensitizing	T790M	Sensitizing	T790M		
*2*	exon 19 del	pos	exon 19 del	pos	needle biopsy	pleura
18	exon 19 del	pos	exon 19 del	pos	cytology	lymph node
34	exon 19 del	pos	exon 19 del	pos	needle biopsy	lung
37	exon 19 del	pos	exon 19 del	pos	needle biopsy	bronchus
14	exon 19 del	**neg** ^**a**^	exon 19 del	**pos**	cytology	pleural fluid
11	L858R	neg	L858R	neg	cytology	lymph node
4	**neg**	neg	**L858R**	neg	cytology	liquor
13	L858R	neg	L858R	neg	cytology	pleural fluid
25	L858R	**pos**	L858R	**neg**	needle biopsy	lung
28	exon 20 ins	neg	exon 20 ins	neg	needle biopsy	lymph node
8	L858R	neg	L858R	neg	cytology	lymph node
20	L858R	neg	L858R	neg	cytology	bronchus/trachea
39	G719C/S768I	**pos**	G719C/S768I	**neg**	biopsy	bronchus
42	exon 19 del	neg	exon 19 del	neg	cytology	vertebral bone
40	exon 19 del	neg	exon 19 del	neg	needle biopsy	lung

*del* deletion, *ins* insertion, *pos* positive, *neg* negative

^a^discordant *EGFR* results between plasma and tissues are indicated in bold letters

All the post-TKI tissue specimens were positive for the original sensitizing *EGFR* mutation and 5 of them displayed the T790M resistance mutation. When the results obtained on tissue specimens were compared with those of the corresponding plasma samples, 4/15 cases resulted discordant (26.7%). The original sensitizing *EGFR* mutation in patient 4 and the T790M resistance mutation in patient 14 were not detected in plasma by tag-based NGS, although they were both present in post-TKI tissue specimens (Table 2). Patient 14 also resulted T790M-negative by ddPCR on cfDNA. In contrast, the remaining discordant cases were found T790M-positive in cfDNA and not in tissues (patients 25 and 39). In both the two post TKI tissues, absence of T790M mutation was confirmed by ddPCR (cut-off > 0.5%; Additional file 3: Table S3). Collectively, the concordance between tissue and plasma was of 93.3% for sensitizing *EGFR* mutations and 80% for the T790M mutation. Considering mutations found in tumor tissues as reference values, the tag-based NGS appeared to have 80% of sensitivity and specificity for the T790M detection in plasma, in line with other reports (Mayo-de-las-Casas et al. 2017).

### Clinical characteristics of the patients with T790M-positive and T790M-negative cfDNA

Clinical data were available for 40/42 patients within the cohort (Additional file 1: Table S1); among these, 18 patients resulted positive for the T790M mutation with tag-based NGS (Additional file 4: Table S4). No significant correlation was observed between T790M status (positive vs. negative) and gender, ECOG (Eastern Cooperative Oncology Group) performance status, smoking habit, age, line of treatment in which EGFR TKI was administered, response to EGFR TKI, or sites of disease progression (extra-thoracic or intra-thoracic) during TKI treatment. However, we observed that positivity for cfDNA T790M mutation was more frequent in patients with the exon 19 mutation than in those with the exon 21 mutation (11/18 vs 5/18, Fisher *p*-value: 0.046). In addition, all T790M mutation-positive patients had received gefitinib, whereas T790M was not found in patients treated with erlotinib or afatinib (Chi Squared *p*-value: 0.013); however, this result might be influenced by the substantial disproportion of the administered TKI which favored gefitinib. Among the 18 T790M-positive patients, 17 received treatment with osimertinib (80 mg/day) and were considered evaluable for clinical outcomes (Table 3).

**Table 3:** Evaluation of response in osimertinib treatment patients according to T790M status by tag-based NGS

Patient	Sex	Age (yrs, median 71)	Sensitizing *EGFR* mut	osimertinib, response	plasma T790M	
					Real Time PCR	tag-based NGS
24	M	58	exon 19 del	PR	pos	pos
27	F	74	L858R	PD	pos	pos
25	F	71	L858R	SD	neg	pos
14	F	75	exon 19 del	SD	neg	neg^a^
35	M	64	exon 19 del	PR	neg	pos
26	F	66	L858R	PR	neg	pos
15	M	75	L858R	SD	neg	pos
2	M	71	exon 19 del	PR	neg	pos
18	F	72	exon 19 del	PR	pos	pos
6	M	78	exon 19 del	PR	pos	pos
10	F	71	exon 19 del	PR	pos	pos
36	F	85	exon 19 del	SD	pos	pos
37	F	65	exon 19 del	PR	pos	pos
39	M	75	G719C	PR	pos	pos
33	F	82	L858R	not evaluable	neg	pos
16	F	66	exon 19 del	SD	neg	pos
22	F	62	exon 20 ins	SD	neg	pos

*del* deletion, *PR* Partial Response, *PD* Progressive Disease, *SD* Stable Disease, *pos* positive, *neg* negative

^a^this patient has been treated with third generation TKI because of T790M-positivity in post-TKI tissue

All but one of these were positive for the T790M mutation by tag-based NGS on plasma, while in one the mutation was observed in tissue specimen. Eight of these 17 patients also were T790M mutation positive by Real Time PCR on plasma.

Fourteen patients were evaluable for objective response assessment by RECIST (Response Evaluation Criteria in Solid Tumors) 1.1 as their CT-scans were available at our Institution (Table 3), while all the 17 patients were evaluable for progression-free survival (PFS) and overall survival (OS). All the patients but one achieved at least disease control as best response. Among the 16 patients who were evaluable for RECIST best objective response, nine achieved partial response (PR, 64.28%), six stable disease (SD, 37.5%) and one patient experienced progressive disease (PD, 6.25%). The waterfall plot for objective response of 14/16 patients is reported in (Additional file 6: Figure S2). Most patients with exon 19 deletions (7/9 cases) achieved objective response with osimertinib compared to those patients with other sensitizing mutations (1/4 cases). The median PFS of the osimertinib-treated patients was 8.8 months and the median OS was 16.7 months. There were no differences between patients with exon 19 deletions and those with other mutations in terms of PFS (8.8 vs. 8.6 months; Log Rank *p*-value: 0.550) or OS (18.0 vs. 16.7 months; Log Rank p-value: 0.513). When we compared the clinical outcome of patients receiving osimertinib according to the results of NGS and Real Time PCR on plasma, we observed the following results. Among the 17 patients who were T790M-positive at tag-based NGS on plasma, eight were positive also at Real Time PCR on plasma, while nine were negative. With regards to RECIST response, T790M Real Time PCR-positive patients achieved the following outcomes: six PR, one SD, one PD; Real Time PCR-negative patients were divided as if follows: three PR, five SD. With regards to survival, among the T790M tag-based NGS-positive patients receiving osimertinib, no significant difference was observed between Real Time PCR-positive and negative patients, both in terms of PFS (12.2 vs. 8.6 months; Log Rank p-value: 0.177) and OS (19.2 vs. 11.6 months; Log Rank p-value: 0.143); similarly to response, these data were based on a small patient population and limited follow up.

## Discussion

To date, the management of NSCLC patients progressing during EGFR TKIs treatment, includes an initial attempt to identify the T790M mutation in the patient’s plasma. In the case of a negative result, a new biopsy or fine-needle aspiration is indicated, when feasible, in order to exclude or confirm the resistance causing mutation (Normanno et al. 2017; Rolfo et al. 2018). This algorithm increases the chances of detecting T790M mutations while reducing the number of biopsies, but may still result in delayed start of subsequent treatments if the mutation is not detected in plasma. Therefore, any improvement of the sensitivity of tests on cfDNA represents a relevant clinical achievement.

Since the discovery of the importance of sensitizing *EGFR* mutations in the pathogenesis and treatment of NSCLC, several different Real Time PCR assays have been employed for the identification of *EGFR* mutations in tissue. When utilized on plasma (Kim et al. 2013; Normanno et al. 2017), it was found that, despite high specificity, these methods were hampered by a low sensitivity. The advent of NGS has opened up new perspectives mostly because of its multiplexed gene approach, although the sensitivity of this method remains challenging, given that a value of 1–5% may be considered an acceptable limit of detection. Attempts to overcome these limits may increase the risks of false positive calls.

The technology used in this study presents several advantages compared to the classical NGS approach, since the molecular tagging step generates single tag DNA molecule that are amplified in a subsequent step. The allelic variants will be called only if two identical molecular tags (referred to as family tags) share the same mutation. The use of single-strand barcodes aided in removing mostly late arising PCR errors as well as sequencer miscalls, while maintaining an optimal sensitivity and specificity. Our analytical tests showed that a 0.1% sensitivity could be readily achieved with 30 ng doses of the standard reference for all *EGFR* allelic variants considered and that this dose could be lowered at 20 ng while maintaining satisfactory results (https://assets.thermofisher.com/TFS-Assets/LSG/brochures/verification-oncomine-lung-cfdna-ion-s5-white-paper.pdf).

In our study, tag-based NGS improved detection rate for both sensitizing and, perhaps more important, T790M resistance mutations compared to Real Time PCR (85.7 and 42.85% versus 61.9 and 21.4%, respectively). The assay was especially sensitive for L858R variation and the T790M resistance mutations that were not detectable by the Real Time PCR and had low allele frequency down to 0.06 and 0.07%, respectively.

Since the T790M mutation is usually present in cfDNA in quantities lower than those of the sensitizing *EGFR* mutations (this study and Del Re et al. 2017; Karlovich et al. 2016), it is justified the need of high sensitivity tests but the disadvantage of increasing false positive signals should be taken into account. However, in this study, the presence of T790M in cfDNA by tag-based NGS was an unlikely finding in the absence of a sensitizing *EGFR* mutation. Therefore, the testing for plasma sensitizing *EGFR* mutation may serve as internal control that informs the likelihood of falsely negative T790M results and concomitantly, provide an indirect proof for circulating tumor derived DNA in the plasma.

Since ddPCR is known together with BEAMing PCR, to have an high technical sensitivity (down to 0.01%), we used it to confirm and validate our tag-based NGS for the only T790M mutation, and interestingly, we found equivalent sensitivity with a 100% concordance and a similar T790M allelic frequency of the positive cases. However, in our opinion even though the ddPCR deliver satisfying analytical and clinical sensitivity, we believe that the amount of cfDNA needed and the single hotspot detected per reaction, may be limiting factors for its routine application.

In our study, the proportion of patients positive for T790M mutation detected in cfDNA by tag-based NGS was in the 50% range, consistently with data reported for post-TKI biopsy tissues (Yu et al. 2013; Hata et al. 2013; Sequist et al. 2011; Oxnard et al. 2011).

Although the concordance for *EGFR* sensitizing mutations was almost complete between cfDNA and post-TKI tissues, some discrepancy was noted for the T790M mutation and in line with previous studies (Kim et al. 2013; Normanno et al. 2017). Indeed, our finding is not surprising and lack of concordance between plasma and post-TKI tissues depends on tumor heterogeneity as well as on specific sites of the tissue biopsy (Ilie & Hofman 2016). Interestingly, we found an enrichment of the T790M mutation among cases with exon 19 deletions. No other characteristics were correlated with the T790M-mutated status, even the thoracic versus extra thoracic metastatic sites, supporting that biological rather than clinical features may have a role in the development of T790M mutation (Ke et al. 2017).

Moreover, the tag-based NGS method appears suitable for the detection of new gene variants conferring resistance to osimertinib such as the C797X mutations as well as other mutations with similar functions (Thress et al., 2015b). It is known that cells with *EGFR* C797S mutation may be still sensitive to first generation TKI (and to osimertinib) when present in trans rather than in a cis configuration with T790M (Niederst et al. 2015; Arulananda et al. 2017). In particular NGS technology compared to standard methods, is able to identify both C797X and T790M mutations on the same amplicon and subsequently their cis or trans configuration. In line with this, two osimertinib treated patients (pts. 24 and 35, Additional file 4: Table S4), progressed under therapy and the tag-based NGS detected a concurrent C797S and C797G resistance mutations in cis configuration (Additional file 7: Figure S3). Therefore this NGS method may prove useful to make further therapeutic decisions.

A limit of our study may be the relative small cohort of patients analyzed, but our patients group is representative of a real life routine in the management of TKI progressed NSCLC patients.

So far, despite NGS still remains a quite expensive method, it may represent a performing test in some diagnostic settings, such as the cfDNA analysis for clinical therapy in advanced NSCLC (Coco et al. 2015). Indeed, firstly NGS technology is highly preferable for the multiplexing ability to parallel screening of different genes. Secondly, NGS approach compared to standard methods, prevents the splitting of a scarce genetic material, such as the cfDNA, in various independent reactions thus reducing additional biases particularly important when low mutational events are investigated.

Lastly, the tag-based NGS technology while increasing sensitivity and concomitantly reducing false positive calls, provide a precise determination of the T790M allelic level helping in the stratification of patients into different groups, that can be subsequently investigated for their osimertinib response (Karlovich et al. 2016; Niederst et al. 2015; Ariyasu et al. 2018).

## Conclusions

The search of *EGFR* T790M mutation in ctDNA rather than in tumor tissue DNA is becoming a reliable alternative procedure. However, ctDNA analysis is technically challenging and consequently, clinical laboratories are required to implement molecular assays in order to provide reliable and accurate *EGFR* test results.

**Fig. 4:**
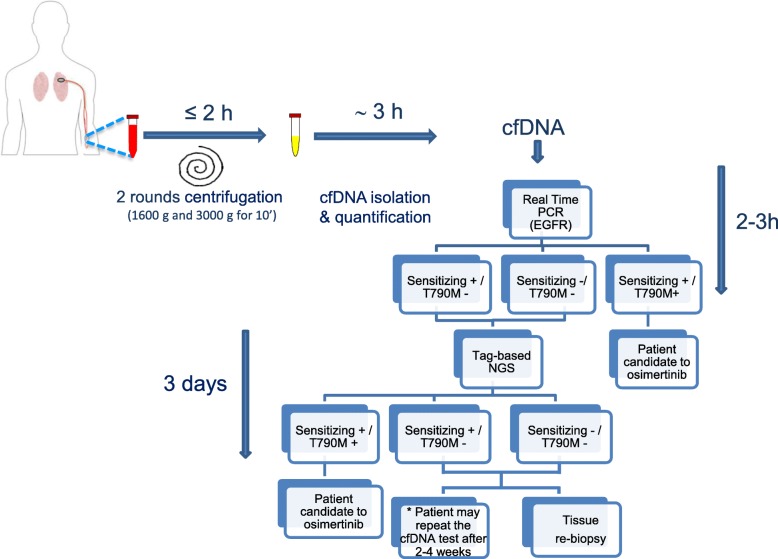
Workflow for the identification of *EGFR* T790M in TKI progressed patients with advanced NSCLC. Procedures and timing of cfDNA *EGFR* testing from sample arrival is represented together with the decision algorithm suggested. *Patients resulting T790M negative after NGS analysis on cfDNA can undergone tumor biopsy, when feasible. Alternatively, the T790M negative patient can be retested on a second cfDNA after 2–4 weeks following the National Scientific Society recommendations on liquid biopsy (https://www.aiom.it/wp-content/uploads/2018/09/2018_biopsialiquida.pdf)

In conclusion this study shows that a tag-based NGS outperformed in ctDNA of post-TKI progressed NSCLC patients compared to Real Time PCR, especially for detection of the resistance T790M mutation. In this context, we propose an algorithm (Fig. 4) that can be applied for the clinical management of TKI progressed patients with advanced NSCLC. Despite the discussed relative high cost of methodology, in some instances tag-based NGS may help in reducing stressful and invasive procedures.

Further larger scale studies are needed to corroborate application of tag molecular sequencing in the search of T790M in a clinical diagnostic context.

## Additional files

Additional file 1: **Table S1.** Clinical characteristics of the patients’ cohort. Samples from 42 patients were available for the analysis; among these, clinical data from 40 patients were available and retrospectively collected. (DOCX 22 kb)

Additional file 2: **Table S2.** List of genes and genomic coordinates of the corresponding hotspots covered by Oncomine™ Lung cfDNA Assay is reported. The Cosmic_ID for gene hotspots are also indicated. (DOCX 16 kb)

Additional file 3: **Table S3.** Cut-off assessment of T790M determination on FFPE tissues by ddPCR. Results on 22 normal FFPE tissues are reported. (DOCX 17 kb)

Additional file 4: **Table S4.** Molecular details of *EGFR* mutational assessment in the cohort of 42 NSCLC patients. Results on *EGFR* assessment in plasma of the 42 patients is shown and detailed. (DOCX 29 kb)

Additional file 5: **Figure S1.** Correlation between amount of total cfDNA yields (pg/mL) and *EGFR*-activating mutated allele fractions tested by tag-based NGS. Each diamond represents one plasma sample. (PDF 86 kb)

Additional file 6: **Figure S2.** Waterfall plot of target lesions shrinkage. The patients receiving osimertinib based upon the presence of the T790M mutation detected on ctDNA by tag-based NGS were evaluated by shrinkage of target lesions. (PDF 137 kb)

Additional file 7: **Figure S3.** Integrative Genomic Viewer visualization of C797S and C797G resistance mutations in cis configuration with T790M in patients 24 and 35. Aligned reads representing the exon 20 amplicon are shown. (PDF 23 kb)

## Abbreviations

BEAMing: Beads, emulsion, amplification and magnetics
cfDNA: Cell-free DNA
ctDNA: Circulating tumor DNA
ddPCR: Droplet digital PCR
dPCR: Digital PCR
FDA: Food and Drug Administration
FFPE: Formalin-fixed paraffin-embedded
hg19: Human genome
IGV: Integrative Genomics Viewer
JSON: JavaScript Object Notation
LOD,: Limit Of Detection
NGS: Next Generation Sequencing
NSCLC: Non Small Cell Lung Cancer
OS: Overall survival
PD: Progressive Disease
PFS: Progression-free survival
PR: Partial Response
RECIST: Response Evaluation Criteria In Solid Tumors
SD: Stable Disease
TKIs: Tyrosine Kinase Inhibitors
TMAP: Torrent Mapping Alignment Program
TVC: Torrent Variant Caller
VCF: Variant Caller Format
