# Supplementary material for: Tag-based next generation sequencing: a feasible and reliable assay for EGFR T790M mutation detection in circulating tumor DNA of non small cell lung cancer patients
Source: Mol Med. 2019 Apr 27;25:15. doi: 10.1186/s10020-019-0082-5 (PMC6487061; doi:10.1186/s10020-019-0082-5)
Supplement: Supplementary file 1 — Table S1. Clinical characteristics of the patients’ cohort. Samples from 42 patients were available for the analysis; among these, clinical data from 40 patients were available and retrospectively collected. (DOCX 22 kb) [file 10020_2019_82_MOESM1_ESM.docx]

| **Additional File 1: Table S1**  Clinical characteristics of the patients’ cohort | |
| --- | --- |
|  | Number (%) |
| **PATIENTS** | |
| Global number | 42 |
| Patients with available clinical data | 40^ |
| **GENDER** | |
| Male | 14 (35.0%) |
| Female | 26 (65.0%) |
| **AGE AT THE TIME OF PLASMA ANALYSIS** | |
| Median | 71 years |
| Range | 48-84 years |
| **SMOKING HABIT** | |
| Never | 23 (57.5%) |
| Former/ current | 17 (42.5%) |
| **HISTOLOGY** | |
| Adenocarcinoma | 40 (100%) |
| **TUMOR STAGE** | |
| IV | 40 (100%) |
| **ECOG PERFORMANCE STATUS** | |
| ECOG= 0 | 10 (25.0%) |
| ECOG= 1 | 25 (62.5%) |
| ECOG= 2 | 5 (12.5%) |
| ***EGFR* MUTATION** | |
| Exon 18 (G719A or G719C) | 2 (5.0%) |
| Exon 19 (deletions) | 18 (45%) |
| Exon 20 (insertions) | 4 (10.0%) |
| Exon 21 (L858R) | 15 (37.5%) **^a^** |
| Exon 21 (L861Q) | 1 (2.5%) |
| **ADMINISTERED EGFR TKI** | |
| Afatinib | 4 (10.0%) |
| Erlotinib | 2 (5.0%) |
| Gefitinib **^b^** | 31 (77.5%) |
| Gefitinib followed by afatinib **^c^** | 2 (5.0%) |
| N/A **^d^** | 1 (2.5%) |
| **LINE OF TREATMENT OF EGFR TKI** | |
| First line | 34 (85.0%) |
| Second line | 3 (7.5%) |
| First and second line **^c^** | 2 (5.0%) |
| N/A **^d^** | 1 (2.5%) |
| **BEST RESPONSE TO EGFR TKI (RECIST 1.1)** | |
| Complete response | 0 (0.0%) |
| Partial response | 18 (45.0%) |
| Stable disease | 15 (37.5%) |
| Progressive disease | 2 (5.0%) |
| N/A **^e^** | 5 (12.5%) |
| **SITE OF DISEASE PROGRESSION** | |
| Intra-thoracic | 13 (32.5%) |
| Extra-thoracic | 27 (67.5%) |

Samples from 42 patients were available for the analysis; among these, clinical data from 40 patients were available and retrospectively collected. **^a^** The two patients not reported in the table were L858R in original tumors and in some results have been counted (see “Clinical characteristics of the patients with T790M-positive and T790M-negative ctDNA”); **^b^** Four patients received gefitinib in combination with vinorelbine as first line within a clinical trial (NCT02319577); **^c^** Two patients received gefitinib in first line and subsequently received afatinib in second line; **^d^** One patient with exon 20 insertion did not receive EGFR TKIs, but underwent mutational analysis on plasma at progression, and was considered evaluable for comparison between tag-based NGS and Real Time PCR; **^e^** One patient did not receive EGFR TKIs (**^d^**), while previous CT-scans for RECIST assessment were not available for 4 patients.
