# Supplementary material for: Tag-based next generation sequencing: a feasible and reliable assay for EGFR T790M mutation detection in circulating tumor DNA of non small cell lung cancer patients
Source: Mol Med. 2019 Apr 27;25:15. doi: 10.1186/s10020-019-0082-5 (PMC6487061; doi:10.1186/s10020-019-0082-5)
Supplement: Supplementary file 2 — Table S2. List of genes and genomic coordinates of the corresponding hotspots covered by Oncomine™ Lung cfDNA Assay is reported. The Cosmic_ID for gene hotspots are also indicated. (DOCX 16 kb) [file 10020_2019_82_MOESM2_ESM.docx]

**Additional file 2: Table S2** Genomic regions covered by the Oncomine™ Lung cfDNA panel

| **Chromosome** | **Genomic coordinates (hg19)** | **GENE_ID** | **COSMIC_ID** |
| --- | --- | --- | --- |
| **1** | 115,256,504-115,258,763 | **NRAS** | COSM583; COSM572; COSM573; COSM574; COSM575; COSM568; COSM24668; COSM569; COSM570; COSM144577; COSM564; COSM565; COSM566; COSM559; COSM560; COSM12723; COSM561; COSM562; COSM563; COSM571 |
| **2** | 29,432,658-29,445,485 | **ALK** | COSM28060; COSM28056; COSM28062; COSM28493; COSM28499; COSM28492; COSM28500; COSM99137; COSM1169447; COSM28491; COSM28054; COSM4381101; COSM53063; COSM28057; COSM28055; COSM28061; COSM28059; COSM28498; COSM1169448; COSM4381100; COSM99136; COSM144252; COSM97185; COSM1407659; COSM98475 |
| **3** | 178,936,066-178,952,102 | **PIK3CA** | COSM760; COSM763; COSM775 |
| **6** | 117,641,093- 117,641,133 | **ROS1** | COSM1072521 |
| **7** | 55,241,670-55,259,533 | **EGFR** | COSM12988; COSM12428; COSM12371; COSM13009; COSM13427; COSM85796; COSM48981; COSM51525; COSM6252; COSM6253; COSM6239; COSM18425; COSM12382; COSM6218; COSM12369; COSM6255; COSM51504; COSM6225; COSM12384; COSM12370; COSM12383; COSM12678; COSM6223; COSM12377; COSM12376; COSM13428; COSM12381; COSM6241; COSM6240; COSM2741500; COSM6213; COSM6224; COSM22943; COSM6227 |
| **7** | 116,411,985-116,423,510 | **MET** | COSM339515; COSM707; COSM24687; COSM48564; COSM35468; COSM29633; COSM698; COSM696; COSM703; COSM598581; COSM1447477; COSM699; COSM690; COSM700; COSM691; COSM694; COSM1568673 |
| **7** | 140,453,089-140,481,425 | **BRAF** | COSM476; COSM470; COSM1133046; COSM459; COSM460; COSM1548505; COSM451 |
| **12** | 25,380,260-25,398,310 | **KRAS** | COSM552; COSM554; COSM553; COSM532; COSM522; COSM527; COSM517; COSM512; COSM521; COSM516; COSM518; COSM520 |
| **15** | 66,727,422-66,774,155 | **MAP2K1** | COSM555604; COSM3503329; COSM1562837; COSM1725008; COSM1235481; COSM4756761; COSM1235478; COSM235614; COSM1315861; COSM1167912; COSM3386991; COSM232755 |
| **17** | 7,574,010-7,579,326 | **TP53** | COSM11411; COSM11287; COSM10660; COSM10704; COSM10659; COSM10749; COSM10779; COSM43896; COSM10743; COSM10656; COSM10817;COSM43871; COSM10725; COSM6549; COSM10810; COSM10834; COSM10785; COSM11081; COSM11196; COSM10662; COSM43947; COSM10758; COSM43687; COSM10687; COSM10648; COSM10670; COSM11148; COSM10714; COSM10808; COSM10645; COSM6815; COSM43559; COSM10889; COSM45940 |
| **17** | 37,880,965- 37,881,007 | **ERBB2** | COSM20959 |

The table shows genomic coordinates of genes investigated by the panel. This includes 11 genes and covers 169 hotspots including single nucleotide variants and short indels frequently mutated in non-small cell lung cancer (NSCLC). COSMIC references are also specified as reported by manufacturer in the BED region file of the Oncomine™ Lung cfDNA panel.
