# Supplementary material for: Tag-based next generation sequencing: a feasible and reliable assay for EGFR T790M mutation detection in circulating tumor DNA of non small cell lung cancer patients
Source: Mol Med. 2019 Apr 27;25:15. doi: 10.1186/s10020-019-0082-5 (PMC6487061; doi:10.1186/s10020-019-0082-5)
Supplement: Supplementary file 3 — Table S3. Cut-off assessment of T790M determination on FFPE tissues by ddPCR. Results on 22 normal FFPE tissues are reported. (DOCX 17 kb) [file 10020_2019_82_MOESM3_ESM.docx]

| **Additional file 3: Table S3** Cut-off assessment of T790M determination on FFPE tissues by ddPCR | | | | |
| --- | --- | --- | --- | --- |
| ***Sample ID*** | **Year** | **T790M copies/µL** | **Wild Type**  **copies/µl** | **T790M Fractional**  **Abundance %** |
| ***4*** | 2005 | 0,21 | 63,0 | 0,33 |
| ***5*** | 2005 | 0,05 | 21,8 | 0,23 |
| ***9_07*** | 2007 | 0,49 | 118,0 | 0,42 |
| ***10*** | 2007 | 0,10 | 59,4 | 0,17 |
| ***11*** | 2007 | 0,34 | 100,5 | 0,23 |
| ***43*** | 2009 | 0,17 | 40,0 | 0,43 |
| ***41*** | 2009 | 0,17 | 43,3 | 0,38 |
| ***36*** | 2009 | 0,18 | 39,3 | 0,45 |
| ***35*** | 2009 | 0,00 | 25,0 | 0,00 |
| ***34*** | 2009 | 0,16 | 57,1 | 0,29 |
| ***33*** | 2009 | 0,16 | 43,3 | 0,37 |
| ***32*** | 2009 | 0,16 | 31,0 | 0,50 |
| ***31*** | 2009 | 0,48 | 98,0 | 0,49 |
| ***30*** | 2009 | 0,07 | 20,2 | 0,36 |
| ***29*** | 2008 | 0,12 | 27,6 | 0,42 |
| ***21*** | 2009 | 0,21 | 54,7 | 0,38 |
| ***1*** | 2017 | 0,15 | 680,0 | 0,02 |
| ***3*** | 2017 | 0,10 | 797,0 | 0,01 |
| ***9_17*** | 2017 | 0,89 | 271,0 | 0,33 |
| ***7*** | 2017 | 0,20 | 789,0 | 0,02 |
| ***8*** | 2017 | 0,15 | 620,0 | 0,02 |
| ***4*** | 2017 | 0,10 | 690,0 | 0,01 |

22 FFPE normal tissues from a tissue bank archival were tested in ddPCR for T790M mutation. False positive detection of T790M ranged from 0.05 to 0.5% and thus cut-off for positivity >0.5%.
