# Supplementary material for: Tag-based next generation sequencing: a feasible and reliable assay for EGFR T790M mutation detection in circulating tumor DNA of non small cell lung cancer patients
Source: Mol Med. 2019 Apr 27;25:15. doi: 10.1186/s10020-019-0082-5 (PMC6487061; doi:10.1186/s10020-019-0082-5)
Supplement: Supplementary file 4 — Table S4. Molecular details of EGFR mutational assessment in the cohort of 42 NSCLC patients. Results on EGFR assessment in plasma of the 42 patients is shown and detailed. (DOCX 29 kb) [file 10020_2019_82_MOESM4_ESM.docx]

| **Additional file 4: Table S4** Molecular details of the EGFR mutational assessment in the cohort of 42 NSCLC patients | | | | | | | | |
| --- | --- | --- | --- | --- | --- | --- | --- | --- |
|  | **Real Time PCR** | | **tag-based NGS** | | | | **ddPCR** |  |
| **pt** | **^a^Sensitizing mutation(s)** | **T790M** | **cfDNA ng/mL plasma** | **cfDNA ng/ reaction** | **^b^*EGFR* mutation(s)** | **VAF (%)** | **T790M (%)** | **T790M/ sens mut ratio** |
|  |  |  |  |  |  |  |  |  |
| **6** | exon 19 del | pos | 10.4 | 16.9 | L747_P753delinsS | 3.59 |  |  |
|  |  |  |  |  | T790M | 2.36 | 2.1 | 0.66 |
| **10** | exon 19 del | pos | 7.04 | 8.58 | L747_P753delinsS | 7.06 |  |  |
|  |  |  |  |  | T790M | 1.71 | 1.1 | 0.24 |
| **18** | exon 19 del | pos | 8.8 | 12.87 | E746_S751delinsA | 2.13 |  |  |
|  |  |  |  |  | T790M | 0.1 | 0.14 | 0.047 |
| **24** | exon 19 del | pos | 7.84 | 19.11 | E746_A750del | 14.38 |  |  |
|  |  |  |  |  | T790M | 9.29 | 9.7 | 0.646 |
| **27** | L858R | pos | 16 | 39 | L858R | 4.9 |  |  |
|  |  |  |  |  | T790M | 0.6 | 1.2 | 0.122 |
| **36** | exon 19 del | pos | 17.44 | 36 | E746_A750del | 2.38 |  |  |
|  |  |  |  |  | T790M | 1.74 |  | 0.73 |
| **37** | exon 19 del | pos | 6.78 | 8.27 | L747_T751del | 0.9 |  |  |
|  |  |  |  |  | T790M | 0.54 | 0.4 | 0.6 |
| **38** | exon 19 del | pos | 16.17 | 30.81 | L747_T751del | 11.11 |  |  |
|  |  |  |  |  | T790M | 2.95 | 4.5 | 0.266 |
| **39** | G719X | pos | 11.04 | 26.91 | G719C | 21 |  |  |
|  |  |  |  |  | T790M | 14.47 |  | 0.69 |
|  |  |  |  |  | S768I | 52 |  |  |
| **2** | exon 19 del | neg | 5.12 | 8.32 | L747_P751del | 4.13 |  |  |
|  |  |  |  |  | T790M | 0.21 | na | 0.05 |
| **15** | L858R | neg | 7.38 | 12 | L858R | 14.58 |  |  |
|  |  |  |  |  | T790M | 0.63 | 0.52 | 0.045 |
| **16** | exon 19 del | neg | 6.56 | 16 | E746_A750del | 0.16 |  |  |
|  |  |  |  |  | T790M | 0.18 | 0.23 | 1.12 |
| **25** | L858R | neg | 2.8 | 6.37 | L858R | 2.17 |  |  |
|  |  |  |  |  | T790M | 0.78 | 0. 7 | 0.36 |
| **33** | L858R | neg | 8.9 | 18.5 | L858R | 1.32 |  |  |
|  |  |  |  |  | T790M | 0.49 | na | 0.378 |
| **35** | exon 19 del | neg | 277.3 | 52 | E746_A750del | 0.1 |  |  |
|  |  |  |  |  | T790M | 0.17 | 0.1 | 1.7 |
| **22** | neg | neg | 4.9 | 8 | A763_Y764insFQEA | 1.7 |  |  |
|  |  |  |  |  | T790M | 0.54 | 0.66 | 0.323 |
| **26** | neg | neg | 3.38 | 8.23 | L858R | 0.75 |  |  |
|  |  |  |  |  | T790M | 0.24 | na | 0.324 |
| **34** | neg | neg | 9.1 | 14.04 | E746_A750del | 0.08 |  |  |
|  |  |  |  |  | T790M | 0.07 | 0.07 | 0.875 |
| **1** | L858R | neg | 10.67 | 26 | L858R | 16.87 |  |  |
| **12** | exon 19 del | neg | 7.1 | 17.29 | E746_S752delinsV | 0.28 | neg |  |
| **13** | L858R | neg | 8.3 | 8 | L858R | 1.41 |  |  |
| **14** | exon 19 del | neg | 66.13 | 49.6 | E746_A750del | 0.26 | neg |  |
| **17** | exon 20 ins | neg | 41.6 | 33.8 | V769_D770insASV | 3.77 |  |  |
| **20** | L858R | neg | 11.31 | 27.56 | L858R | 6.08 | neg |  |
| **23** | exon 19 del | neg | 43.79 | 49.26 | L747_A750delinsP | 15.52 | neg |  |
| **31** | L858R | neg | 10.29 | 25.09 | E709G | 25.02 |  |  |
|  |  |  |  |  | L858R | 31.3 |  |  |
| **40** | exon 19 del | neg | 16.7 | 40.7 | E746_A750del | 9.54 | neg |  |
| **41** | exon 19 del | neg | 12.9 | 31.46 | E746_A750del | 1.1 | neg |  |
| **42** | exon 19 del | neg | 5.03 | 11.44 | E746_S752del | 0.51 | neg |  |
| **3** | neg | neg | 5.65 | 13.78 | L858R | 0.16 |  |  |
| **7** | neg | neg | 9.52 | 15.47 | L858R | 0.06 | neg |  |
| **8** | neg | neg | 23.36 | 48.18 | L858R | 0.1 | neg |  |
| **11** | neg | neg | 39.8 | 37.32 | L858R | 0.08 |  |  |
| **19** | neg | neg | 11.2 | 27.3 | L858R | 0.11 |  |  |
| **21** | neg | neg | 6.9 | 14 | E709G | 1.37 | neg |  |
|  |  |  |  |  | G719A | 1.4 |  |  |
| **28** | neg | neg | 5.65 | 13.78 | V769_D770insASV | 1.09 |  |  |
| **4** | neg | neg | 35.84 | 44.8 | no variant |  |  |  |
| **5** | neg | neg | 11.36 | 18.46 | no variant |  |  |  |
| **9** | neg | neg | 9.78 | 14.3 | no variant |  |  |  |
| **29** | neg | neg | 5.33 | 6.5 | no variant |  |  |  |
| **30** | neg | neg | 27.79 | 52.1 | no variant |  |  |  |
| **32** | neg | neg | 92.27 | 43.25 | no variant |  |  |  |

The results on EGFR assessment and the plasma details of 42 post TKI NSCLC patients are shown

^a^ Real Time PCR assay does not distinguish between the different deletions and/or insertions that can be found in EGFR exons 19 and 20, respectively, as well as the various G719 codons, thus only nonspecific mutational informations are provided

^b^ Tag-based NGS is able to identify the various types of exon 19 deletions and exon 20 insertions as well the specific G719 codons and therefore mutations features are reported

VAF, variant allele frequency; na, not assessable
