# Supplementary material for: Tag-based next generation sequencing: a feasible and reliable assay for EGFR T790M mutation detection in circulating tumor DNA of non small cell lung cancer patients
Source: Mol Med. 2019 Apr 27;25:15. doi: 10.1186/s10020-019-0082-5 (PMC6487061; doi:10.1186/s10020-019-0082-5)

Additional file 5: Figure S1 Correlation between amount of total cfDNA yields (pg/mL) and the EGFR-activating mutated allele fractions tested by tag-based NGS

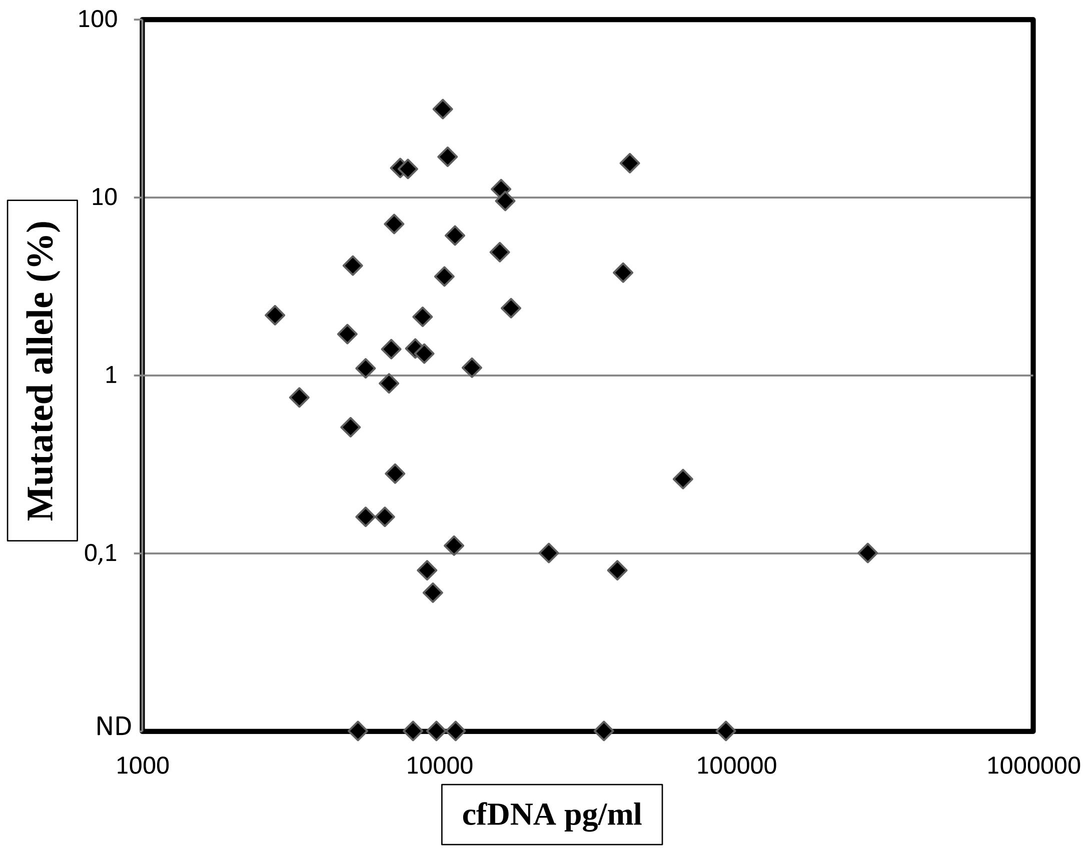

Supplement: Supplementary file 5 — Figure S1. Correlation between amount of total cfDNA yields (pg/mL) and EGFR-activating mutated allele fractions tested by tag-based NGS. Each diamond represents one plasma sample. (PDF 86 kb) [file 10020_2019_82_MOESM5_ESM.pdf]
