# Supplementary material for: Tag-based next generation sequencing: a feasible and reliable assay for EGFR T790M mutation detection in circulating tumor DNA of non small cell lung cancer patients
Source: Mol Med. 2019 Apr 27;25:15. doi: 10.1186/s10020-019-0082-5 (PMC6487061; doi:10.1186/s10020-019-0082-5)
Supplement: Supplementary file 6 — Figure S2. Waterfall plot of target lesions shrinkage. The patients receiving osimertinib based upon the presence of the T790M mutation detected on ctDNA by tag-based NGS were evaluated by shrinkage of target lesions. (PDF 137 kb) [file 10020_2019_82_MOESM6_ESM.pdf]

Additional file 6: Figure S2 Waterfall plot of target lesions shrinkage

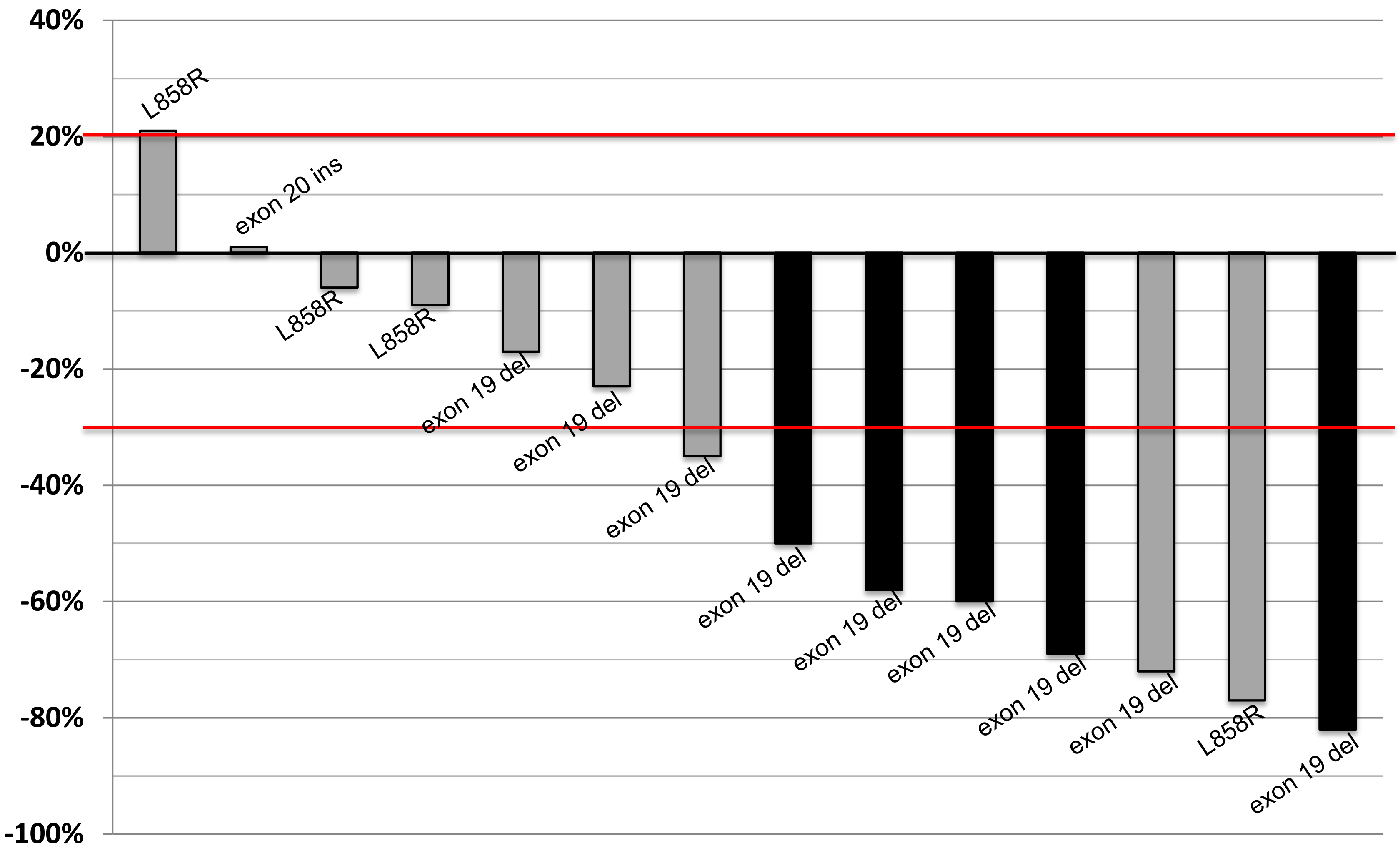

Shrinkage of target lesion in evaluable patients receiving osimertinib based upon the presence of the T790M mutation detected on ctDNA by tag-based NGS is shown. Cut-offs for progressive disease (+20%) and partial response (-30%) are indicated as per RECIST 1.1. The original sensitizing mutation of each patient is shown (black and grey histograms indicate T790M-positive and T790M-negative patients by Real Time PCR, respectively) . del, deletions; ins, insertions.
