# Supplementary material for: Tag-based next generation sequencing: a feasible and reliable assay for EGFR T790M mutation detection in circulating tumor DNA of non small cell lung cancer patients
Source: Mol Med. 2019 Apr 27;25:15. doi: 10.1186/s10020-019-0082-5 (PMC6487061; doi:10.1186/s10020-019-0082-5)
Supplement: Supplementary file 7 — Figure S3. Integrative Genomic Viewer visualization of C797S and C797G resistance mutations in cis configuration with T790M in patients 24 and 35. Aligned reads representing the exon 20 amplicon are shown. (PDF 23 kb) [file 10020_2019_82_MOESM7_ESM.pdf]

Additional file 7: Figure S3 Integrative Genomic Viewer visualization of C797S and C797G resistance mutations in cis configuration with T790M in patients 24 and 35

patient 24

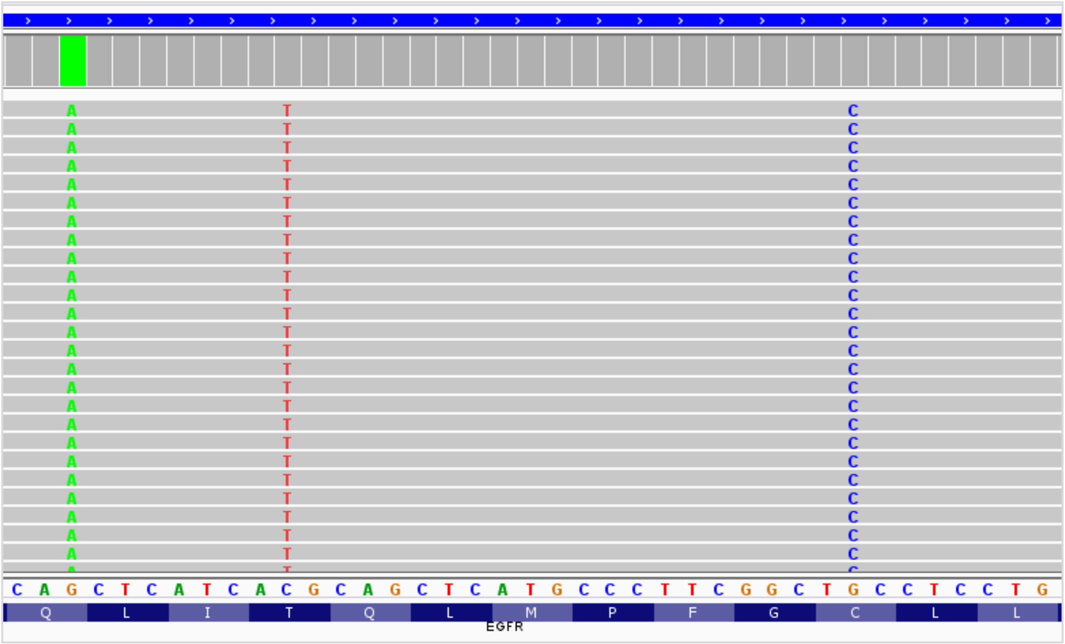

patient 35

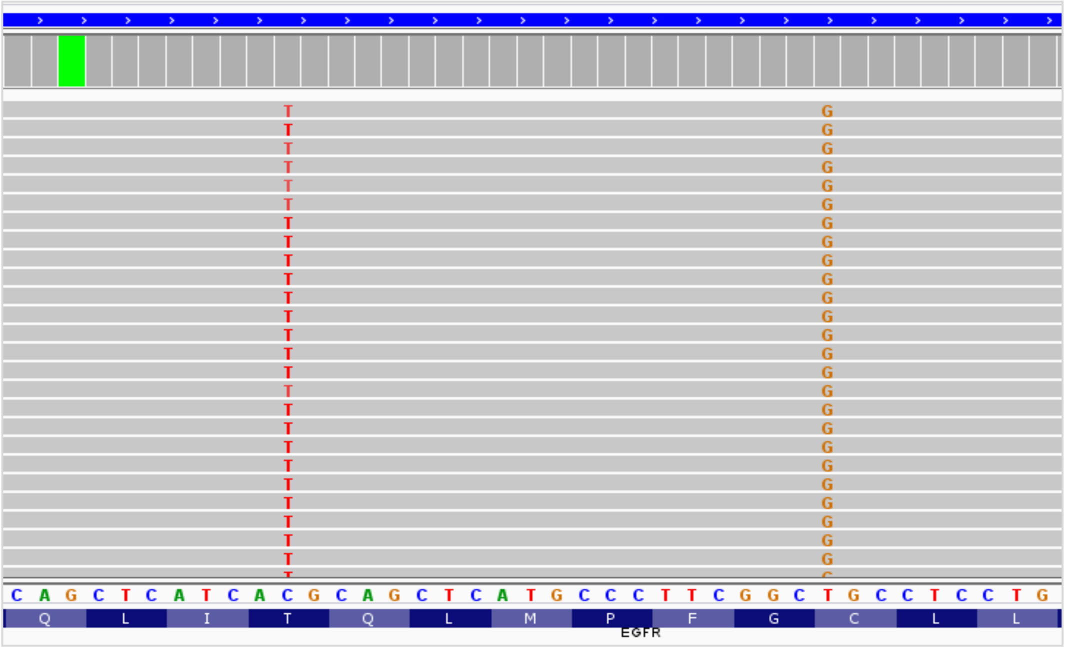

Deep sequencing data on the two patients showing the *EGFR* T790M and C797X mutation detected in cis allelic relationship
